# Cost-effectiveness of clostridial collagenase ointment on wound closure in patients with diabetic foot ulcers: economic analysis of results from a multicenter, randomized, open-label trial

**DOI:** 10.1186/s13047-015-0065-x

**Published:** 2015-02-28

**Authors:** Travis A Motley, Adrienne M Gilligan, Darrell L Lange, Curtis R Waycaster, Jaime E Dickerson

**Affiliations:** University of North Texas Health Sciences Center, Bone and Joint Institute, Fort Worth, TX USA; Smith & Nephew Inc., 3909 Hulen Street, Fort Worth, TX 76107 USA; Department of Pharmacotherapy, University of North Texas Health Sciences Center, Fort Worth, TX USA; Department of Cell Biology and Anatomy, University of North Texas Health Sciences Center, Fort Worth, TX USA; Department of Pediatrics, University of North Texas Health Sciences Center, Fort Worth, TX USA

**Keywords:** Clinical outcome, Collagenase, Cost-effectiveness, Debridement, Diabetes, Foot ulcer, Health resource utilization, Economic outcome, Wound healing

## Abstract

**Background:**

Approximately 10%–15% of people with diabetes develop at least one foot ulcer during their lifetime. Treatment of diabetic foot ulcers (DFUs) represents a significant economic burden. Enzymatic debridement with clostridial collagenase ointment (CCO) can be used to remove necrotic tissue from wounds. This study examined the impact of CCO as an effective adjunct therapy to serial sharp debridement (SSD) and assessed the cost-effectiveness of CCO compared with standard DFU treatments over 1 year.

**Methods:**

Adults 18 years or older with a diagnosis of type 1 or type 2 diabetes who had a neuropathic DFU were enrolled in a 12-week, randomized, open-label trial. Patients were randomly assigned to either treatment with CCO + SSD or to investigator-selected supportive care + SSD (Control). A 3-state Markov model with a 1-week cycle length was developed using wound-closure rates from the trial to estimate the number of healed-wound weeks and the expected DFU cost per patient. The 3 states included unhealed, healed, and death. Results were extrapolated to 1 year to estimate the number of healed-wound weeks per treatment and the average cost to achieve epithelialization. The perspective of the analysis was that of the payer, specifically, the third party payer.

**Results:**

The study sample included 55 patients (28 in CCO group; 27 Control). The majority were men (74.5%) with a mean age of 57.9 years. Projected healing rates were greater for the CCO + SSD group compared to Control (89% vs. 80%, respectively). The expected number of epithelialized weeks accumulated over 1 year was 25% greater in the CCO + SSD group than for Control (35 vs. 28 weeks, respectively). Over a 1-year time horizon, the expected cost per DFU was greater in the Control group than the CCO group ($2,376 vs. $2,099, respectively). The estimated cost per ulcer-free week was 40% higher for Control ($85/closed-wound week) than for CCO + SSD ($61/closed-wound week).

**Conclusions:**

CCO + SSD therapy is a cost-effective method of debridement in the management of patients with DFUs, providing better outcomes at a lower cost. Further high quality trials are needed to confirm this finding.

**Trial registration:**

This study was registered at ClinicalTrials.gov as NCT01408277.

**Electronic supplementary material:**

The online version of this article (doi:10.1186/s13047-015-0065-x) contains supplementary material, which is available to authorized users.

## Background

More than 23 million people (approximately 8% of the population) in the United States (US) currently have diabetes mellitus [1]. Among these individuals, approximately 1%–4% will develop a diabetic foot ulcer (DFU) annually, and 10%–15% will develop a DFU in their lifetime [2-4]. DFUs are often refractory to therapy and can be associated with substantial medical complications, such as osteomyelitis and lower extremity amputation (LEA). According to systematic literature reviews of DFUs from 1980 to 2004, between 70,000 and 80,000 patients with diabetes have an amputation every year in the US [1,5] with resultant escalating comorbidities, additional amputation, and increased risk of mortality [5,6]. The 1-year mortality rate after LEA in people with diabetes ranges from 10% to 50%, and the 5-year mortality rate after LEA is 30%–80% [7-9]. In addition, DFU represents a significant economic burden, accounting for 20%–40% of health resource utilization spent on diabetes management [3]. The mean annual direct treatment costs of DFU per patient are approximately $20,000 [10-13], and costs for treating a DFU 2 years after diagnosis has increased from $45,301 (in 2008 US dollars) to $49,209 in 2013 [14]. As healthcare costs continue to increase at an exponential rate, it is important that clinicians understand the cost-effectiveness of the care that they are administering so treatments that provide increased clinical benefit and are considered cost-effective can be utilized [15].

Currently, the 3 major components in treating DFU are debridement, offloading, and infection control. Debridement is generally believed to be a critical factor in proper wound management [16,17]. Debridement means “to unleash”. The generally accepted immediate goal of debridement is the removal of debris and nonviable tissue as a means to the end goal of unleashing the capacity of the wound to heal itself. Depending on the method, debridement may also permit thorough evaluation of wound extent; reduce contamination by pathogens and biofilm-forming bacteria; and create a wound edge composed of cells that have the ability to respond to molecular signals [18,19]. Serial sharp debridement (SSD) is generally considered the “gold standard” method for wound debridement [2,16,17,19]. However, SSD is generally acknowledged to be a relatively nonselective method of debriding a wound because normal tissue is also unavoidably removed. A systematic review of the literature has concluded that although the rationale for using SSD to remove devitalized, necrotic tissue and expose healthier tissue seems logical, the evidence for its role in enhancing healing is deficient [20]. Several studies have suggested that SSD may work in synergy with other treatment approaches, such as growth factors, cell therapy, or other methods of debridement (i.e. enzymatic) [16,21-23].

Serial or maintenance debridement of DFU is necessary whenever devitalized tissue is present [2,19,24]. Alternative methods of debridement that remove necrotic tissue while sparing healthy tissue may be advantageous and decrease the frequency of, or the need for, repeat SSD. One such approach is enzymatic debridement using clostridial collagenase ointment (CCO) [25,26]. In patients with pressure ulcers, CCO as formulated in Santyl® ointment (Smith & Nephew Inc., Fort Worth, Texas) has been shown to provide complete and effective debridement of ulcers in 85% of patients by Day 42 of treatment without initial or concomitant SSD [27]. CCO applied daily to a DFU provides ongoing debridement of the ulcer, and therefore, establishes a wound environment conducive to healing. In a randomized, parallel-group, open-label, multicenter, 12-week clinical study comparing CCO alone and saline-moistened gauze with SSD, only CCO treatment resulted in a statistically significant mean percent decrease from baseline in wound area at the end of treatment and at the end of follow-up (*p* = 0.01 and 0.01, respectively) [21]. When assessing CCO utilization in the US, a pharmacoepidemiology analysis of 96 hospital-based outpatient wound centers revealed that, of 21,677 DFUs treated from 2007–2012, approximately 17% received CCO [28].

Only minimal research has been done to assess the impact of enzymatic debridement as an effective adjunct therapy to SSD. Given the recent trends in cost containment for health care systems, it is important for health care providers and coverage decision makers to compare the initial cost of advanced therapies to the overall total cost per episode of care when deciding the appropriate allocation of resources. Therefore, the objectives of this study were to assess the cost-effectiveness of CCO compared with standard DFU treatments over a span of 1 year.

## Methods

### Study participants

Adults 18 years or older with a diagnosis of type 1 or type 2 diabetes requiring medications to normalize blood glucose levels were evaluated for eligibility to participate in the trial. Eligible participants had to have neuropathic foot ulcers from 0.5 cm^2^ to 10 cm^2^ in area for a minimum of 30 days. Key eligibility criteria included that participants have adequate arterial blood flow as evidenced by an ankle brachial index (ABI) of >0.7 and ≤1.1, be able to follow instructions and perform dressing changes at home or have a caregiver willing to perform dressing changes according to the protocol, and be willing to use an appropriate offloading device when necessary to keep weight off of the foot ulcer. Alternatively, if ABI was unable to be measured, a Doppler waveform consistent with adequate blood flow to the region of the foot with the target ulcer (biphasic or triphasic waveforms) was considered acceptable. Patients who had an infection with systemic toxicity; cellulitis associated with the foot ulcer; lymphangitic streaking; deep tissue abscess; gangrene; an infection of the muscle, tendon, joint or bone; foot ulcer tunneling; or an ulcer on the heel or over a Charcot deformity that could not be offloaded were excluded from participation in the trial. The trial was performed in compliance with the ethical principles of the Declaration of Helsinki and Good Clinical Practice. The trial protocol and participant consent forms were reviewed and approved by an accredited Institutional Review Board [Protocol No: 017-101-09-030, Sterling Institutional Review Board, Atlanta, GA, USA]; and all participants provided written informed consent before participating in the trial.

### Study design and intervention

**Figure 1:**
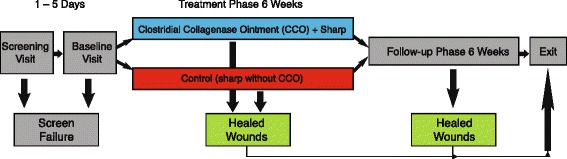
**Study schematic.** Reproduced with permission from *Wounds* [29].

Clinical findings and methods for this economic analysis were previously published by Motley et al. [29]. The trial was a US-based, prospective, randomized, parallel-group, open-label (non-blinded), multicenter, 12-week clinical study, carried out at 7 outpatient sites in 5 states (Arizona, 1 site; California, 1 site; Michigan, 1 site; Texas, 3 sites; and Virginia, 1 site). The trial consisted of a 6-week treatment phase and a 6-week follow-up phase (Figure 1). Fifty-five study participants were recruited from August 29, 2011 to October 02, 2012. The primary objective of the trial was to compare the mean percent wound area change from baseline over the 6-week treatment period, and at the end of the follow-up period in patients receiving daily application of CCO (Santyl® ointment) plus sharp SSD (CCO + SSD) *versus* Investigator-selected supportive care (i.e. silver dressings and hydrogels) plus SSD (Control). Patients were randomly assigned to either CCO + SSD (n = 28) or Control (n = 27) for the 6-week treatment phase. Treatment was given for 6 weeks and patients were followed for up to an additional 6 weeks or to complete wound closure, whichever occurred first. During the 6-week follow-up phase all ulcers (in both treatment groups) that had not closed received daily dressing changes consisting of a foam primary dressing and a single layer cast padding held in place with a self-adherent bandage. All patients agreed to wear an offloading boot or other appropriate device.

Patients in the CCO + SSD group had CCO applied once daily, approximately 2 mm thick, to the foot ulcer. The wound was gently covered with an Allevyn® nonadhesive dressing (Smith & Nephew, Hull, United Kingdom), which was maintained in place with a secondary bandage (Coban®, 3 M, St. Paul, Minnesota). Patients in the Control group were treated with DFU standard treatments based on the investigators’ clinical preferences. Investigator-selected treatment regimens were allowed in order to emulate ‘real-world’ clinician practice patterns and preferences. These treatments included wet-to-dry dressings (n = 5), hydrogel (n = 1), silver dressing (n = 12), silver sulfadiazine cream (n = 5), or alginate dressing (n = 4), in addition to SSD. Silvercel® (Systagenix Wound Management, Gargrave, United Kingdom) was the primary silver dressing used by investigators in the clinic and was used as the reference price (in the cost analysis) for silver dressings. Hyperbaric or negative pressure therapy was not allowed. At the investigator’s discretion, a hydrogel could be used if deemed necessary to maintain a moist wound environment. Wound area was measured at each study visit using the Applied Research Associated New Zealand Ltd (ARANZ) Silhouette™ digital image capture and wound measurement device (ARANZ Medical, Christchurch, NZ). All patients received SSD commencing with the Week 1 visit. Patients in both groups received SSD of the target ulcer at each of the scheduled study visits (Weeks 2 through 12) if any of the relevant subscales (Edges, Undermining, Necrotic Tissue Type, or Necrotic Tissue Amount) of the Wound Assessment Tool were ≥ 3. If the relevant subscales were all ≤ 2, then SSD was not performed. The Wound Assessment Tool is a modification of the Bates-Jensen tool [30] and provides a standardized, numerical score consisting of 8 subscales in the assessment of the health and overall condition of the wound. Although this was an open-label study, allocation to intervention group was determined using a blinded (or centralized) randomization sequence to prevent potential bias resulting from subjectivity in allocation to treatment (i.e. allocation concealment).

### Economic analysis

A Markov model was developed to compare the cost and outcomes of CCO + SSD versus Control using the wound closure rates from the clinical trial to estimate the number of healed wound weeks and the expected DFU cost per patient. Outputs from the Markov model were then used to derive a cost-effectiveness ratio for each treatment group. Using this approach, results were extrapolated to 1 year to estimate the number of closed-wound weeks per treatment as well as the average cost to achieve epithelialization. CCO utilization was derived based upon the manufacturer’s recommended dosing algorithm [31] using the wound surface area sizes from the CCO cohort. For this analysis, it was assumed that wounds were cleaned and dressed daily as they were in the clinical trial. Over 6 weeks the average CCO utilization in the clinical trial was 9.5 grams. Because the clinical trial lasted 12 weeks, 1 tube of CCO was used in the primary analysis. However, a sensitivity analysis using 2 tubes was analyzed to assess changes in expected total costs of care over 1 year. Markov models are well suited to aid decision-making in clinical situations when events and costs transition over time [32]. The Markov model was developed using TreeAge Pro (TreeAge Pro version 2013, TreeAge Software Inc., Williamstown, Massachusetts).

### Model inputs

#### Time horizon

A 1-year time horizon was chosen to allow sufficient time for wound closure in both groups. Costs associated with outpatient treatment continued to accrue until epithelialization was reached.

#### Three-state markov model and transition probabilities

**Figure 2:**
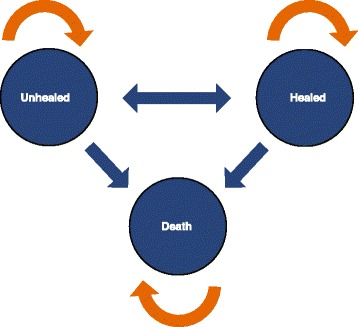
**Three-state diagram of Markov model.**

A 3-state Markov model with a cycle length of 1 week was chosen to follow the unhealed, healed, and death stages of a DFU (Figure 2). Since results were extrapolated out to 1-year, death was included in order to capture the overall mortality rate of the U.S. population for individuals 35 years of age and older. State 1, the unhealed state, represents a healing DFU and, consequently, all the costs associated with treatment in the outpatient setting (i.e. sharp debridement, enzymatic debridement, and clinic visits). State 2, the epithelialized phase, represents a closed wound requiring no further dressing or treatment and, consequently, incurring no further costs. State 3, the death state, was defined as a probability of death (per week) set to 0.000147 on the basis of Centers for Disease Control data for 2010 annual mortality rate (all causes) for persons ages 35 and older (the ages of patients in the clinical trial) [33].

The transition probabilities from the unhealed phase to the epithelialized phase were determined using wound-closure rates from the clinical trial. At the end of 12 weeks, approximately 65% of patients in the CCO + SSD group were considered healed compared to 47% in the Control group. These probabilities were extrapolated to 52 weeks assuming cumulative probability rates over time using equations described by Briggs et al. [34-36] Additional file 1: Figure S1]. Using this time-dependent Markov model provides a robust method to modeling a chronic illness, since the assumption of constant transition probabilities is considered too restrictive for applications in healthcare [36]. The probability of ulcer recurrence was used the Persson et al. model for DFUs [37]. The probability of ulcer recurrence from the Persson et al. model was estimated by a Markov model of diabetic neuropathic lower extremity ulcers developed by Abt Associates, Inc. (Abt Inc., Cambridge, MA) [38]. These weekly transition rates [Table 1] were used to populate the Markov model and to determine the clinical and economic outcomes. Weekly transition rates incorporated all daily dressings and cleanings that occurred throughout the week. Infection rates were considered for the Markov analysis. Although a few patients who had an infection were admitted to their local inpatient facilities, the number was small and was similar between the CCO and Control groups; therefore, because the hospitalization rates were similar and would not significantly affect the cumulative costs of care for either treatment, they were omitted from the model input.

**Table 1:** **Percent of occurrence and transition probabilities**

**Parameter**	**Percent**	**Weekly transition probability**
CCO + SSD healing rate*		
Weeks 1, 6, 9, and 11	5.0	0.05
Weeks 2, 4, and 12	10.0	0.10
Weeks 3, 5, 7, 10, and 19-52	0.0	0.00
Week 8	15.0	0.15
Weeks 13-18	6.9	0.07
Control healing rate*		
Weeks 1-2, 4, 6-7, 9, 11, and 26-52	0.0	0.00
Week 3	15.0	0.15
Weeks 5 and 10	5.0	0.05
Weeks 8 and 12	11.0	0.11
Weeks 13-25	5.6	0.06
**Ulcer recurrence** ^**†**^		
Weeks 1-52	47.1	0.01
**All-cause mortality** ^**‡**^	0.0147	<0.01

CCO, clostridial collagenase ointment; SSD, serial sharp debridement.

*NCT01408277, Briggs et al. [31-33].

^†^Persson et al. [34].

^‡^Centers for Disease Control for 2010 for persons 35–95 years [30].

#### Clinical outcomes definition

The clinical benefit for the Markov model was defined as “epithelialized weeks” and represents the expected number of weeks that the wound was closed over the 1-year time horizon. This was presented as ulcer-free weeks to effectively demonstrate the differences in the wound healing trajectories between the 2 treatment groups. Ulcer-free weeks represent the average expected time, in weeks, that DFUs remain closed in the 2 comparative cohorts given their respective transition probabilities from the unhealed state to the epithelialized (healed) state. Ulcer-free weeks are the mathematical complement of open wound weeks and represent a positive measure of clinical outcome.

#### Economic outcome definition

The perspective of the analysis was that of the payer, specifically, the third-party payer. Given that the cost of care for Medicare beneficiaries with a DFU exceeds $33,000 annually for total reimbursement of all Medicare services [39], the third-party payer of interest was the Centers for Medicare and Medicaid Services (CMS). The CMS maximum-allowable costs were used as proxies for assessing total cumulative cost of care. Only the direct medical costs of care were considered in the economic analysis. All costs were reported in 2013 US dollars, and no discounting of costs was utilized because the duration of the model was 1 year. A cost-effectiveness analysis was performed assessing cost per epithelialized week on a per-patient basis. Derivation of costs is displayed in Table 2. Costs for SSD, enzymatic debridement, dressings, wrappings, medications and evaluation and management (E/M) visits (levels 2 and 3 for physicians and level 2 for facility) were used for the first 12 weeks per the design of the clinical trial. After 12 weeks, both treatment arms assumed weekly E/M visits (level 1 for physician and level 1 for facility). A level 1 E/M visit controls for a weekly dressing change, assuming the patient did not achieve complete wound closure. The costs of offloading were not included in this analysis. CMS does not cover reimbursement of offloading devices for the treatment of DFU, except the Total Contact Cast (TCC) [40]. Since TCC was not used in this trial, costs for offloading devices were not included in the economic model as they are a direct cost to the patient.

**Table 2:** **Unit cost table**

**Category**	**Item**	**Current procedural terminology/diagnosis-related group code**	**Medicare costs**
Clinic visits^*†^	Physician, SSD	97597	$23.48
	Facility, SSD	97597	$106.96
	Facility, enzymatic debridement	97602	$71.54
	Physician, clinic visit (level 1)	99211	$8.85
	Facility, clinic visit (level 1)	99211	$56.77
	Physician, clinic visit (level 2)	99212	$24.50
	Facility, clinic visit (level 2)	99212	$73.68
	Physician, clinic visit (level 3)	99213	$49.67
Dressings^‡^	Silvercel	A6196^§^	$7.96 each
	Alginate	A6196^§^	$7.96 each
	Allevyn® nonadhesive	A6209^§^	$8.09 each
	Hydrogel sheeting dressing	A6242^§^	$7.17 each
	Wet-to-dry gauze	A6402^§^	$0.13 per yard
	4x4 gauze	A6402^§^	$0.13 per yard
Wrappings^¶^	Coban®	A6454^§^	$0.84 per yard
	Coflex	A6454^§^	$0.84 per yard
Medication	Collagenase ointment (CCO)	J3590^§^	$182.76 per tube
	Silver sulfadiazine 1% cream	591081055^‖^	$9.94 per tube

CCO, clostridial collagenase ointment; DFU, diabetic foot ulcer; SSD, serial sharp debridement.

^*^”Clinic” refers to a hospital-based outpatient wound care department.

^†^If any type of debridement occurred, then no clinic visit billing was allowed. If there was no debridement, the clinic visit codes were used because dressings were changed frequently. When enzymatic debridement was performed, only the facility was reimbursed.

^‡^Only Allevyn® non-adhesive was used in patients who received CCO. Other dressings were considered standard of care in the treatment of DFU.

^§^Healthcare Common Procedure Coding System code.

^¶^Each dressing was wrapped after applying CCO or standard-of-care dressing.

^‖^National drug code.

#### Sensitivity analyses

All sensitivity analyses were performed in TreeAge Pro (TreeAge Pro version 2013, TreeAge Software Inc., Williamstown, Massachusetts). Sensitivity analysis is the process of changing the value of an input parameter to assess the magnitude of its effect on the final results of the analysis. This type of analysis tests the robustness of the models assumptions (i.e. variables chosen for model input) on the results. Deterministic sensitivity analysis provides an explanation for the source of ranges used, along with justification for choice of the variables included. In this analysis, the probability of healing for CCO and Control; the costs of SSD, enzymatic debridement and clinic visits; and the probability of ulcer recurrence were included in the sensitivity analyses. Clinical trials invariably include wounds with a large variety of sizes and shapes. Given the uncertainty that exists in rates of healing with wounds of various sizes and shapes [41], a ±50% compared with the base case was utilized to increase the robustness of the model’s results. In addition, the frequency of dressing changes was varied from twice daily to once every 3 days; and the amount of CCO utilization was varied from 1 to 2 tubes. Outputs were displayed in a tornado diagram for one-way analyses. A tornado diagram graphically depicts changes in results relative to changes in model input assumptions and ranks these changes according to their magnitude. Variables with substantial uncertainty or expectation of sensitivity were selected for these analyses.

A probabilistic sensitivity analysis was performed to evaluate parameter uncertainty by using second-order Monte-Carlo simulations of 10,000 trials in which all model inputs were varied simultaneously. This method is comprised of generating a “dummy” data set by resampling with replacement (i.e. randomly selecting 1 patient at a time) from the original data set and repeating this random patient selection until the dummy data set reaches the same size as the original [32].

## Results

A total of 55 participants were included in this study. Participant’ and wound characteristics are displayed in Table 3. The majority of the participants were men (74.5%), and the mean age of the sample was 57.9 years (standard deviation [SD] = 12.3). Nearly all of the participants were under 85 years (94.5%), White/Caucasian (85.5%) and non-Hispanic/Latino (83.6%). The average wound surface area was 1.9 cm^2^ (SD = 1.4), and the mean ankle brachial index was 1.0 (SD = 0.1). The majority of wounds were plantar (78.2%) or plantar/medial (10.9%) and located on the left foot (56.4%). For the 6-week treatment phase, the average number of SSDs was 4.1 (SD = 1.8), and decreased during the 6-week follow-up phase (3.1, SD = 2.3). Wounds treated with CCO + SSD decreased in area from an average of 1.9 cm^2^ at baseline to 0.6 cm^2^ at the end of the treatment phase (*p* < 0.001). Wounds in the Control group went from an average area of 1.8 cm^2^ to 1.2 cm^2^, which was not statistically significant (*p* = 0.31). At the end of the follow-up phase, average wound area was 0.8 cm^2^ in the CCO + SSD group (*p* < 0.001 from baseline) and 1.2 cm^2^ in the Control group.

**Table 3:** **Patient demographics and wound characteristics**

	**Total**	**CCO**	**Control**	***p*** **-value**
**Demographic**	**(n = 55)**	**(n = 28)**	**(n = 27)**	**ANOVA/Chi-square**
**Age**				
Mean ± SD	57.9 ± 12.3	56.9 ± 12.0	59.0 ± 12.7	0.54
Minimum, Maximum	35.0, 97.0	35.0, 97.0	39.0, 88.0	
**Geriatric, n (%)**				
<85 years	52 (94.5)	27 (96.4)	25 (92.6)	0.53
≥85 years	3 (5.5)	1 (3.6)	2 (7.4)	
**Sex, n (%)**				
Men	41 (74.5)	21 (75.0)	20 (74.1)	0.94
Women	14 (25.5)	7 (25.0)	7 (25.9)	
**Race, n (%)**				
White/Caucasian	47 (85.5)	25 (89.3)	22 (81.5)	0.41
Black/African American	8 (14.5)	3 (10.7)	5 (18.5)	
**Ethnicity, n (%)**				
Hispanic/Latino	9 (16.4)	5 (17.9)	4 (14.8)	0.76
Non-Hispanic/Latino	46 (83.6)	23 (82.1)	23 (85.2)	
**Ankle brachial index, n (%)**				
n*	33 (60.0%)	16 (57.1)	17 (63.0)	0.52
Mean ± SD	1.0 ± 0.1	1.0 ± 0.1	1.0 ± 0.1	
Minimum, Maximum	0.8, 1.1	0.8, 1.1	0.8, 1.1	
**Wound area (cm** ^**2**^ **)**				
Mean ± SD	1.9 ± 1.4	2.0 ± 1.1	1.8 ± 1.6	0.73
Minimum, Maximum	0.1, 7.5	0.1, 4.6	0.2, 7.5	
**Wound location, n (%)**				
Distal	2 (3.6)	1 (3.6)	1 (3.7)	0.38
Lateral	3 (5.5)	2 (7.1)	1 (3.7)	
Lateral/dorsal/distal	1 (1.8)	0 (0.0)	1 (3.7)	
Plantar	43 (78.2)	20 (71.4)	23 (85.2)	
Plantar/medial	6 (10.9)	5 (17.9)	1 (3.7)	
**Wound side, n (%)**				
Left foot	31 (56.4)	14 (50.0)	17 (63.0)	0.33
Right foot	24 (43.6)	14 (50.0)	10 (37.0)	
**Number of sharp debridements**				
**6 Treatment weeks**				
Mean ± SD	4.05 ± 1.83	4.07 ± 1.88	4.04 ± 1.81	0.45
Minimum, Maximum	1.0, 6.0	1.0, 6.0	1.0, 6.0	
**6 Follow-up treatments**				
N	39 (70.9%)	20 (71.4%)	19 (70.4%)	
Mean ± SD	3.11 ± 2.32	3.32 ± 2.24	2.89 ± 2.45	0.81
Minimum, Maximum	0.0, 6.0	0.0, 6.0	0.0, 6.0	

ANOVA, analysis of variance; CCO, clostridial collagenase ointment; SD, standard deviation.

*Not all patients had an ankle brachial index assessment.

**Figure 3:**
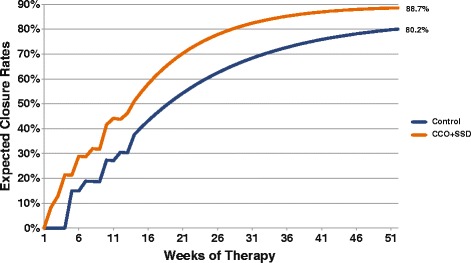
**Expected wound closure rates for CCO + SSD and control treatments.** CCO, clostridial collagenase ointment; SSD, serial sharp debridement.

**Figure 4:**
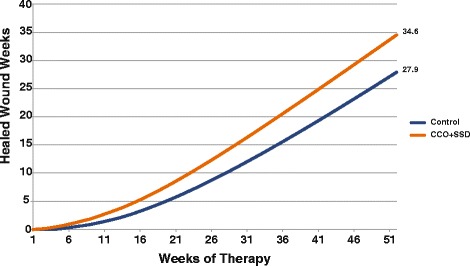
**Healed wound weeks among patients treated with CCO + SSD and control.** CCO, clostridial collagenase ointment; SSD, serial sharp debridement.

Expected wound closure rates between CCO + SSD and Control are displayed in Figure 3. Controlling for death rates and ulcer recurrence over 1 year, the projected healing rates were greater for the CCO + SSD group (89% vs. 80%, respectively). The primary clinical outcome for this economic analysis was closed-wound weeks. On the basis of the transition rates of the prospective clinical trial data previously published by Motley et al. [29], the expected number of epithelialized weeks accumulated over 1 year was 25% greater in the CCO + SSD group compared with the Control group (35 vs. 28 weeks, respectively; Figure 4). To provide another perspective, the clinical compliment to epithelialized weeks (i.e. closed-wound weeks) is open-wound weeks. Consequently, the number of expected open-wound weeks for the CCO + SSD and Control cohorts is estimated at 17 and 24 weeks, respectively. That is, patients receiving the Control intervention would have, on average, 7 additional open-wound weeks (approximately 2 extra months) compared with patients treated with enzymatic debridement as an adjunct to SSD.

**Figure 5:**
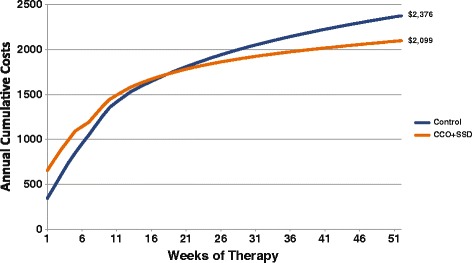
**Cumulative costs for CCO + SSD and control treatments.** CCO, clostridial collagenase ointment; SSD, serial sharp debridement.

A 1-year time span was selected to adequately model the total costs across the entire episode of care for both treatment groups. One year was selected in order to capture the additional costs of closure that occur past the 12 weeks of the study. However, it should be noted that during the 12-week clinical trial, CCO had higher rates of healing, better outcomes, and approximately the same cost compared with Control. The differences in the epithelialization rates between CCO and Control as an adjunct to SSD led to differences in cost between the 2 therapies. Once the DFU epithelializes, wound care therapy is essentially complete and no further costs accrue, unless there is recurrence. The analysis indicated that the expected costs per DFU at the end of the study (i.e., 12 weeks) were $1,580 and $1,530 for the CCO + SSD and Control groups, respectively. However, given the difference in the wound closure trajectories between CCO + SSD and Control, the expected cost (2013 US dollars) per DFU over a 1-year time horizon was $2,099 in the CCO + SSD group and $2,376 in the Control group, a difference of $277 (Figure 5). When CCO utilization was increased to 2 tubes, the expected cost of care over 1-year rose by only $66 (from $2,099 to $2,165), still providing a cost savings of $211 compared with the cost in the Control group.

When the total costs of DFU care were estimated over the course of 1-year, CCO + SSD therapy provided better clinical outcomes at a lower cost of care relative to Control therapy. Patients treated with Control incurred total treatment costs that were approximately 10% higher than those receiving CCO + SSD. The clinical benefit of CCO + SSD was approximately 25% greater than for Control. The estimated cost per ulcer-free week was 40% higher for Control ($85/closed-wound week) than for CCO + SSD ($61/closed-wound week).

### Sensitivity analyses

One-way sensitivity and probabilistic sensitivity analyses were performed on the Markov model parameters to determine the effects of uncertainty on the model assumptions. All model parameters were varied by ±50%, with the exception of the frequency of dressing changes, which were varied from twice daily to once every 3 days. The one-way deterministic sensitivity analyses revealed no thresholds where CCO + SSD lost economic dominance. The tornado diagram shows which variables exerted the most influence on outcomes (Additional file 2: Figure S2). The most influential variables were frequency of dressing changes and the facility cost for sharp debridement. One of the largest varying costs in DFU therapy is the cost/frequency of dressing changes. The assumption is that the clinical outcomes remain the same with costs varying because of the frequency of dressing changes. Rates of healing for patients treated with CCO + SSD and ulcer recurrence also exerted influence on outcomes. CCO utilization had no substantial influence on outcomes. Results from the probabilistic sensitivity analysis (i.e., Monte Carlo Simulation) indicated that costs for CCO + SSD were lower compared with Control. CCO + SSD had the highest average effectiveness of 35 ulcer-free weeks (SD = 1), while the Control group had fewer ulcer-free weeks (28 weeks, SD = 2) (Additional file 3: Table S1).

## Discussion

DFUs often require substantial healing time and are associated with increased risk for infections and other events that can result in severe and costly outcomes [42]. The economic burden of DFU can be explained by multiple factors, including late management of diabetes, high recurrence and amputation rates, complexity of treatment modalities on patients with osteomyelitis, and high morbidity and mortality rates after amputation [43]. The results of this Markov analysis demonstrate that, although CCO is more expensive compared with pharmacoloigcally inert dressings used in the Control group, enzymatic debridement with CCO as an adjunct to SSD can accelerate wound closure, thus reducing the overall cost of DFU care. Findings from the current investigation indicate how the addition of CCO to DFU treatment can reduce the total direct cost of DFU care to the payer. Although the cost of a 30-gram tube of CCO was almost 3 times the cost of Control dressings, the therapeutic effect of CCO + SSD was approximately 25% greater than for Control when wound closure was measured across the entire episode of care.

Annual healthcare costs associated with chronic wound treatment in the U.S. approaches $33 billion [44,45]. Overall, the U.S. spends twice as much per capita as the United Kingdom, Sweden and the Netherlands, yet these countries achieve better overall health outcomes [46]. In 2006 the Tax Relief and Health Care Act was passed, which authorized the establishment of a pay-for-performance program known as Physician Quality Reporting Initiative wherein payment is linked to whether the clinician performs certain tasks in a given time frame for specific patients [47]. Currently there is a Physician Quality Reporting Initiative measure relating to diabetic “foot care”, specifically, performing peripheral neuropathy evaluation and prescribing appropriate footwear [47]. However, offloading of an existing DFU is not a Physician Quality Reporting Initiative measure. As the population ages and the prevalence of diabetes and obesity increase it will be important for third-party payers, such as Medicare and Medicaid, to evaluate the efficacy and effectiveness of treatment practice patterns and Physician Quality Reporting Initiative measures in wound care.

Treatment with CCO + SSD provided 35 ulcer-free weeks compared with 28 weeks in the Control cohort. Stated differently, CCO + SSD had an average expected time to closure of 17 weeks compared with 24 weeks in the Control cohort. These 7 additional open-wound weeks can have substantial clinical, economic, and humanistic impact. The longer a wound remains unhealed, the greater the risk for infection, decreased health-related quality of life, and higher cost of care. Infection control is of utmost importance in DFU treatment because infection is strongly associated with amputation. Lavery et al. prospectively determined risk factors for infection and found that wounds that penetrated the bone had a greater than 30 day duration and a traumatic etiology, were recurrent, occurred in patients with peripheral vascular disease, and had a significantly higher increased risk of infection [48]. Closing the wound approximately 2 months earlier using CCO as an adjunct to SSD could help reduce downstream implications and costs of care for DFU. In addition, the overall cost of care for the CCO + SSD cohort yielded a cost savings of $277 (US dollars) compared with Control. These results demonstrate the economic value of CCO for the treatment of DFUs in outpatient settings.

Per the inclusion/exclusion criteria of this study, the majority of wounds fell into a specific size range; that is, wound area was ≥ 0.1 cm^2^ and ≤ 7.5 cm^2^. In a large retrospective study of 26,599 diabetic wounds, 60% had a mean wound area of > 0.5 cm^2^ and < 7 cm^2^ [49]; therefore, ulcer size in this study is applicable to other DFU patient populations. Regarding age, sex, and race/ethnicity, patients in this study are generalizable to the overall DFU population [50,51].

A limited number of economic analyses comparing advanced therapies with other standards of care exist (particularly within DFU patient populations) [21,52-55]; and results of these analyses are consistent with the current study’s clinical and cost-effectiveness findings. Tallis et al. found that CCO therapy resulted in a higher likelihood of wound surface area reduction, significantly better response rates compared with standard of care DFU therapy, and lower direct average costs per responder in the hospital outpatient department setting over 12 weeks compared with saline-moistened gauze/SSD ($1,607 versus $1,980 US dollars, respectively) among patients with DFUs [21]. Another study by Waycaster et al. using randomized clinical trial data from a long-term care setting showed that CCO had fewer expected wound days (48 versus 147, respectively) at substantially lower cost ($2,003 versus $5,480, respectively) compared with autolytic debridement using a hydrogel dressing for the treatment of pressure ulcers over a 1-year time period [55].

Certain limitations should be considered when interpreting our findings. First, the clinical data used in this analysis were derived from a clinical trial conducted in 7 outpatient sites in 5 states with a relatively small patient sample (55 patients); therefore, the results cannot necessarily be generalized to other dressings, health care settings, or to wounds of other etiologies. Second, this investigation was unblinded, so ascertainment bias cannot be ruled out. However, an open-label (non-blinded) trial was chosen intentionally to allow clinically relevant standard care in the control group, with allocation bias and assessment bias controlled through appropriate randomization and allocation concealment, and the use of a wound measurement device (ARANZ). Third, investigators were only permitted to conduct a SSD if one or more of the subscale parameters of Edges, Undermining, Necrotic Tissue Type, or Necrotic Tissue Amount of the Wound Assessment Tool was ≥ 3. This requirement did not allow for an independent assessment of the relative frequency of SSD for CCO versus Control, as similarity in wound assessment scores necessarily resulted in similarity in use of SSD. Nevertheless, because the frequency of SSD was essentially the same between the 2 treatment groups, the frequency of debridement may be treated as a constant, and therefore, the different outcomes for the 2 groups can be ascribed to the actual treatments used (ie, CCO or the various Control regimens). However, after consultation with wound care specialists, debridement would be conducted in those situations (i.e. undermining, predominance of fibrous or necrotic tissue). Therefore, although debridement was protocol-driven in this study, it is not expected that clinicians would debride differently in a clinical setting. Results indicate that SSD used in conjunction with CCO provides faster healing than SSD used with passive supportive therapies.

## Conclusions

The results of this study demonstrate that CCO is a cost-effective adjunct therapy to SSD. DFUs treated with CCO + SSD yielded almost 2 extra ulcer-free months while costing approximately $300 less than Control. In addition, DFU patients treated with CCO + SSD showed an enhanced rate of healing during the treatment period, and in the follow-up period when cessation of treatment occurred compared to Control.

Enzymatic debridement of DFU with CCO as an adjunct to SSD offers better value compared with various commonly used nonenzymatic supportive care regardless of the outpatient care setting. CCO + SSD therapy is a cost-effective method of debridement in the management of patients with DFU, providing better outcomes at a lower cost of care. Health care providers should consider CCO when using SSD in the treatment of DFUs, although further high quality trials are required to confirm our findings.

## Additional files

Additional file 1: Figure S1. Equations for Event Rate and Transition Probability Calculations. Additional file 2: Figure S2. Parameters affecting cost of wound closure in patients with DFUs. (CCO, clostridial collagenase ointment; DFU, diabetic foot ulcer). Additional file 3: Table S1. Monte Carlo simulation results.

## Abbreviations

ANOVA: Analysis of variance
CCO: Clostridial collagenase ointment
DFU: Diabetic foot ulcer
E/M: Evaluation and management
LEA: Lower extremity amputation
SD: Standard deviation
SSD: Serial sharp debridement
US: United States
