# Erratum to: Cost-effectiveness of clostridial collagenase ointment on wound closure in patients with diabetic foot ulcers: economic analysis of results from a multicenter, randomized, open-label trial

**DOI:** 10.1186/s13047-016-0160-7

**Published:** 2016-08-04

**Authors:** Travis A. Motley, Adrienne M. Gilligan, Darrell L. Lange, Curtis R. Waycaster, Jaime E. Dickerson

**Affiliations:** 1 University of North Texas Health Sciences Center, Bone and Joint Institute, Fort Worth, TX USA; 2 Smith & Nephew Inc., 3909 Hulen Street, Fort Worth, TX 76107 USA; 3 Department of Pharmacotherapy, University of North Texas Health Sciences Center, Fort Worth, TX USA; 4 Department of Cell Biology and Anatomy, University of North Texas Health Sciences Center, Fort Worth, TX USA; 5 Department of Pediatrics, University of North Texas Health Sciences Center, Fort Worth, TX USA

## Erratum

Following publication of the original article [1] it was brought to our attention that some of the data within the article required correction.

Hence, please note that in the second paragraph of the section headed ‘Three-state markov model and transition probabilities’ there is a sentence which reads as:

“At the end of 12 weeks, approximately 65 % of patients in the CCO + SSD group were considered healed compared to 47 % in the Control group.”

However, this should read as:

“At the end of 12 weeks, approximately **47 %** of patients in the CCO + SSD group were considered healed compared to **32 %** in the Control group.”

This alteration does not affect the methodology or results.
