# Supplementary figures and images for: Cost-effectiveness of clostridial collagenase ointment on wound closure in patients with diabetic foot ulcers: economic analysis of results from a multicenter, randomized, open-label trial
Source: J Foot Ankle Res. 2015 Feb 28;8:7. doi: 10.1186/s13047-015-0065-x (PMC4357050; doi:10.1186/s13047-015-0065-x)

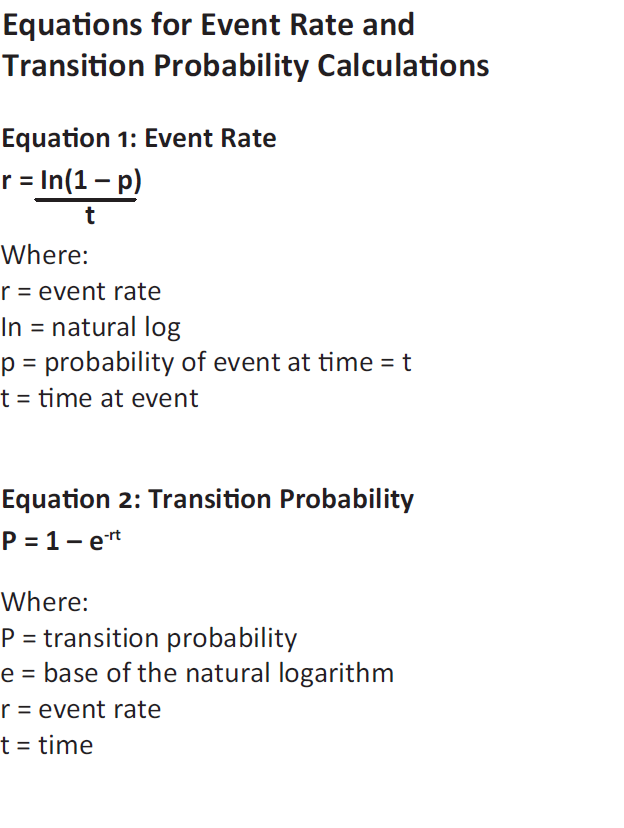

Supplement: Additional file 1: Figure S1. — Equations for Event Rate and Transition Probability Calculations. [file 13047_2015_65_MOESM1_ESM.png]

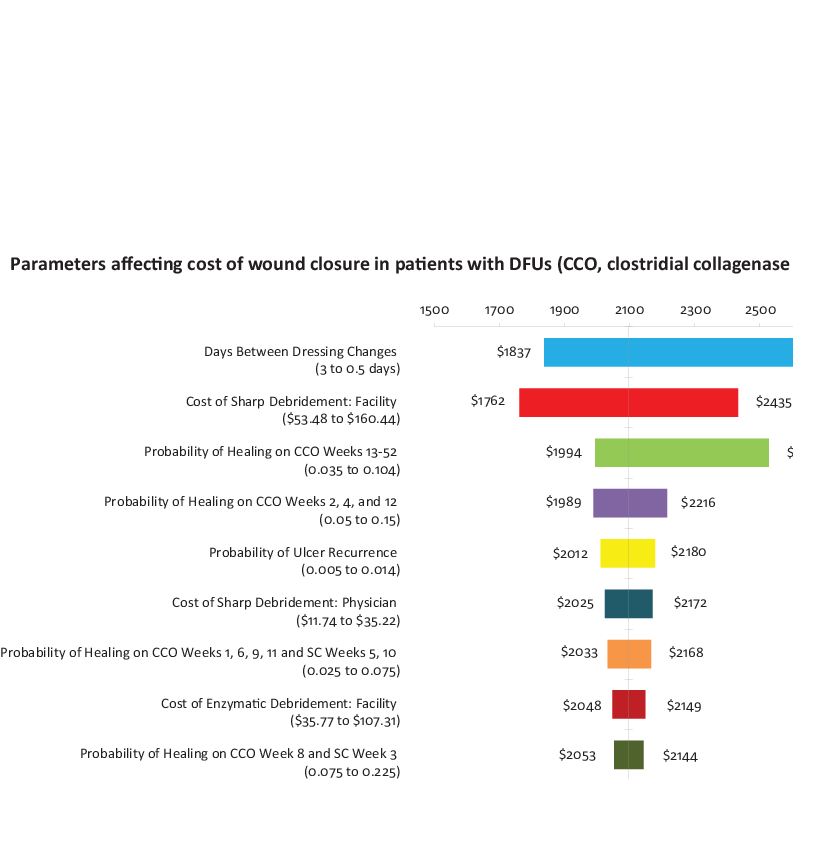

Supplement: Additional file 2: Figure S2. — Parameters affecting cost of wound closure in patients with DFUs. (CCO, clostridial collagenase ointment; DFU, diabetic foot ulcer). [file 13047_2015_65_MOESM2_ESM.png]
