# Supplementary material for: Cost-effectiveness of clostridial collagenase ointment on wound closure in patients with diabetic foot ulcers: economic analysis of results from a multicenter, randomized, open-label trial
Source: J Foot Ankle Res. 2015 Feb 28;8:7. doi: 10.1186/s13047-015-0065-x (PMC4357050; doi:10.1186/s13047-015-0065-x)
Supplement: Additional file 3: Table S1. — Monte Carlo simulation results. [file 13047_2015_65_MOESM3_ESM.pdf]

Supplementary Table 1. Monte Carlo simulation results*

**Results**	**CCO + SSD**	**Control**
Effectiveness mean	35	28
Effectiveness SD	1	2
Effectiveness minimum	29	20
Effectiveness 2.5 percentile	32	24
Effectiveness median	35	28
Effectiveness 97.5 percentile	37	32
Effectiveness maximum	40	35
Cost mean	$2,098	$2,381
Cost SD	$382	$273
Cost minimum	$776	$1,387
Cost 2.5 percentile	$1,455	$1,884
Cost median	$2,061	$2,367
Cost 97.5 percentile	$2,971	$2,964
Cost maximum	$4,405	$3,606

CCO, clostridial collagenase ointment; SD, standard deviation; SSD, serial sharp debridement

*Effectiveness measure was ulcer-free weeks.
